# Supplementary material for: Antimicrobial and Immunomodulatory Potential of Cow Colostrum Extracellular Vesicles (ColosEVs) in an Intestinal In Vitro Model
Source: Biomedicines. 2022 Dec 15;10(12):3264. doi: 10.3390/biomedicines10123264 (PMC9775086; doi:10.3390/biomedicines10123264)
Supplement: Supplementary file 1 [file biomedicines-10-03264-s001.zip › Figure S1.pdf]

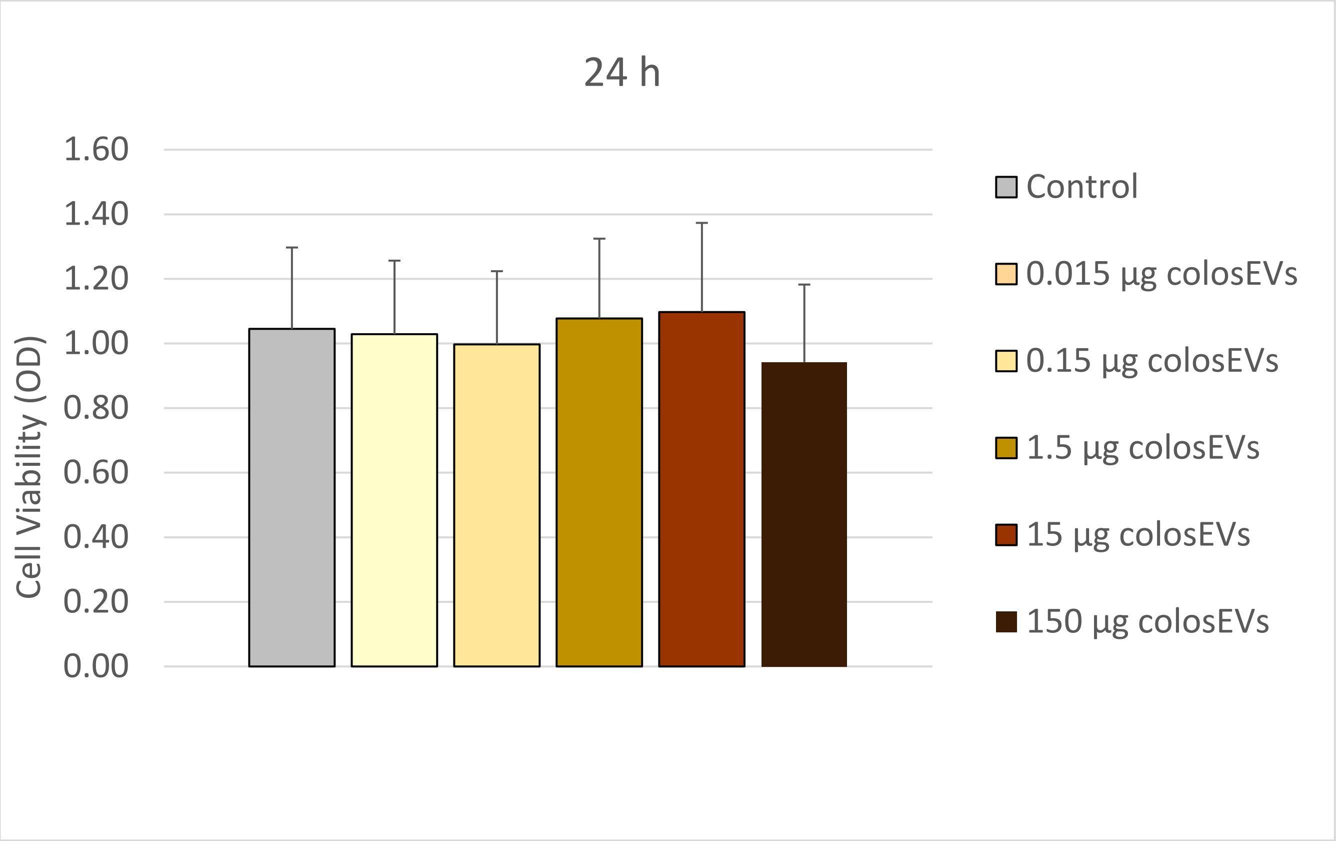


**Figure S1.** Viability of IPEC-J2 after 24 h colosEVs exposure. The different concentrations of colosEVs did not determine a significant difference in terms of cell viability after 24 h. Data are expressed as optical density (OD) ± SD. Differences were evaluated through the Kruskal-Wallis test and applying the post-doc Dunn's Multiple Comparison Test.
