# Supplementary material for: Antimicrobial and Immunomodulatory Potential of Cow Colostrum Extracellular Vesicles (ColosEVs) in an Intestinal In Vitro Model
Source: Biomedicines. 2022 Dec 15;10(12):3264. doi: 10.3390/biomedicines10123264 (PMC9775086; doi:10.3390/biomedicines10123264)
Supplement: Supplementary file 1 [file biomedicines-10-03264-s001.zip › Table_S1.pdf]

Table S1. Primers, amplicon sizes and thermal profiles used for Multiplex and Simplex PCR

Primers	Sequences	Amplicon Size	Cycles	Thermal profiles
eae	For – 5′–CATTGATCAGGATTTTTCTGGTGATA-3'<br>Rev-5′-CTCATGCGGAAATAGCCGTTA-3'	101	45	10 minutes at 45 °C, 10 minutes at 95 °C then 45 cycle, 15s of denaturation and 60s of extension. Acquisition of fluorescence
eae Probe	p-ATAGTCTCGCCAGTATTCGCCACCAATACC			
STb	For – 5′–TGCCTATGCATCTACACAAT-3'<br>Rev-5′-CTCCAGCAGTACCATCTCTA-3'	113		
STa	For – 5′–CAACTGAATCACTTGACTCTT-3'<br>Rev-5′-TTAATAACATCCAGCACAGG-3'	158	30	60s of denaturation at 90°C, 60s of annealing at 55°C, 60s of extension at 70 °C and the final extension of 10 minutes at 70 °C
K99	For – 5′–AATACTTGTTCGGGAGAAA-3'<br>Rev-5′-AACTTTGTGGTTAACTTCCT-3'	230		
CNF1	For – 5′–AGGAAGTTATATTTCCGTAGG-3'<br>Rev-5′-GTATTTGCCTGAACCGTAA-3'	552	35	60s of denaturation at 94°C, 60s of annealing at 63°C, 60s of extension at 72 °C and the final extension of 10 minutes at 72 °C
