# Supplementary material for: Antimicrobial and Immunomodulatory Potential of Cow Colostrum Extracellular Vesicles (ColosEVs) in an Intestinal In Vitro Model
Source: Biomedicines. 2022 Dec 15;10(12):3264. doi: 10.3390/biomedicines10123264 (PMC9775086; doi:10.3390/biomedicines10123264)
Supplement: Supplementary file 1 [file biomedicines-10-03264-s001.zip › Table_S2.pdf]

Table S2: Classes of antibiotic drugs employed for antimicrobial characterization and related clinical breakpoints.

Antibiotic Class	Antimicrobial agent	Clinical Breakpoint (µg/mL)			Source
		S	I	R	
Amphenicols	Florphenicol (FFC)	4	8	16	CLSI Vet 08
Aminopenicillin	Amoxicillin/Clavulanic Acid (AMC)	8/4	16/8	32/16	CLSI Vet 08
	Ampicillin (AMP)	0.25	0.5	1	CLSI Vet 08
Aminoglycosides	Gentamycin (GEN)	4	8	16	CLSI-H M100
	Kanamycin (KAN)	16	32	64	CLSI-H M100
	Aminosidine (AM)	8	16	32	CASFM
1st-2nd gen. Cephalosporines	Cefazoline (CEZ)	2	4	8	CLSI Vet 08
Quinolones	Flumequine (FLQ)	4	8	16	CASFM
Fluoroquinolones	Enrofloxacin (ENRO)	0.25	0.5-1	2	CLSI VET 08
Polymyxin	Colistin (COL)	2	-	4	EUCAST-11
Sulphonamides	Trimethoprim/Sulfamethoxazole (SX-T)	2/38	-	4/76	CLSI Vet 08
	Sulfisoxazole (S)	256	-	512	CLSI-H M100
Tetracyclines	Tetracycline (T)	4	8	16	CLSI VET 08
