# Supplementary material for: Antimicrobial and Immunomodulatory Potential of Cow Colostrum Extracellular Vesicles (ColosEVs) in an Intestinal In Vitro Model
Source: Biomedicines. 2022 Dec 15;10(12):3264. doi: 10.3390/biomedicines10123264 (PMC9775086; doi:10.3390/biomedicines10123264)
Supplement: Supplementary file 1 [file biomedicines-10-03264-s001.zip › Table_S3.pdf]

Table S3: Results obtained from Multiplex PCR and Simplex PCR.

**Target**	**Strain n° 1**	**Strain n°2**
K99	negative	negative
eae	**positive**	**positive**
Sta	negative	negative
STb	negative	negative
CNF1	negative	**positive**
