# Supplementary material for: Antimicrobial and Immunomodulatory Potential of Cow Colostrum Extracellular Vesicles (ColosEVs) in an Intestinal In Vitro Model
Source: Biomedicines. 2022 Dec 15;10(12):3264. doi: 10.3390/biomedicines10123264 (PMC9775086; doi:10.3390/biomedicines10123264)
Supplement: Supplementary file 1 [file biomedicines-10-03264-s001.zip › Table_S4.pdf]

Table S4: MIC values (μg/mL) and related SIR results of antimicrobial agents tested against *E. coli* strains.

**Antimicrobial agent**	**Strain n° 1**		**Strain n°2**	
	MIC values (μg/mL)	SIR results	MIC values(μg/mL)	SIR results
Ampicillin (AMP)	32	I	32	R
Amoxicillin/ Clavulanic Acid (AMC)	8	S	8	S
Aminosidine (AM)	32	R	4	S
Flumequine (FLQ)	4	S	128	S
Cefazoline (CEZ)	8	R	2	S
Colistin (COL)	0.5	S	0.5	S
Enrofloxacin (ENRO)	0,25	S	0.03	S
Florphenicol (FFC)	8	I	4	S
Gentamycin (GEN)	2	S	0,5	S
Kanamycin (KAN)	32	R	4	S
Sulfisoxazole (S)	128	S	256	R
Trimethoprim/Sulfamethoxazole (SX-T)	0.06	S	0.12	S
Tetracycline (T)	2	S	2	S

S: sensitive; I: intermediate; R: resistant
